# Relationship between N95 Amplitude of Pattern Electroretinogram and Optical Coherence Tomography Angiography in Open-Angle Glaucoma

**DOI:** 10.3390/jcm9123854

**Published:** 2020-11-27

**Authors:** Taekjune Lee, Du Ri Seo, Joo Yeon Kim, Wungrak Choi, Sang Yeop Lee, Jun Mo Lee, Gong Je Seong, Chan Yun Kim, Hyoung Won Bae

**Affiliations:** 1 Institute of Vision Research, Department of Ophthalmology, Yonsei University College of Medicine, 50-1, Yonsei-ro, Seodaemun-gu, Seoul 03722, Korea; angelua@naver.com (T.L.); drseo@yuhs.ac (D.R.S.); drjykim@yuhs.ac (J.Y.K.); wungrakchoi@yuhs.ac (W.C.); yeopy@yuhs.ac (S.Y.L.); gjseong@yuhs.ac (G.J.S.); kcyeye@yuhs.ac (C.Y.K.); 2 Department of Ophthalmology, Yongin Severance Hospital, Yonsei University College of Medicine, 363, Dongbaekjukjeon-daero, Giheung-gu, Yongin-si 16995, Korea; 3 Siloam Eye Hospital, 181, Deungchon-ro, Gangseo-gu, Seoul 07668, Korea; eyejune@siloam.co.kr

**Keywords:** pattern electroretinogram, optical coherence tomography angiography, open-angle glaucoma, N95 amplitude

## Abstract

Purpose: The pattern electroretinogram (PERG) is useful to detect retinal ganglion cell (RGC) damage in patients with glaucoma. Optical coherence tomography angiography (OCTA) measures retinal vessel density (VD), which is known to be reduced in glaucoma. There may be correlations between parameters of the PERG and OCTA in open-angle glaucoma (OAG). Methods: In total, 95 eyes of 95 OAG patients and 102 eyes of 102 normal controls were included in this study. N35, P50, and N95 latency along with P50 and N95 amplitude were obtained using the PERG. Retinal VD was measured around the peripapillary and macular area according to the ETDRS grid (concentric circles with diameters of 1, 3, and 6 mm), which is named a center (≤1 mm), an inner (1–3 mm), an outer (3–6 mm), and a full (≤6 mm) area. Pearson correlation analysis was done between parameters, and partial correlation analysis was done after adjusting confounding factors. Results: P50 amplitude, N95 amplitude, and VD of most measured areas were significantly lower in the OAG group compared to the normal group. N95 amplitude showed a statistically significant correlation with parameters of optical coherence tomography and visual field, peripapillary outer and full VD, and macular outer and full VD even after adjusting confounding factors. There was no significant correlation between parameters in the normal group. Conclusions: N95 amplitude was associated with structural and functional change including VD reduction in OAG. Microvascular alterations may be associated with dysfunctional changes of RGC recorded by the PERG in OAG.

## 1. Introduction

Glaucoma is characterized by progressive optic neuropathy with characteristic loss of optic nerve fibers and retinal ganglion cells (RGCs) [1]. Optical coherence tomography (OCT) and visual field (VF) have been standard methods to evaluate structural and functional change for decades. Due to the complex mechanism of the disease and difficulty in establishing a single examination tool with adequate levels of sensitivity and specificity, newer methods have been developed and applied to detect and assess glaucomatous change.

The pattern electroretinogram (PERG) has been investigated in a number of studies with glaucoma [2,3,4,5]. The PERG has the potential to detect dysfunctional RGCs without structural axonal loss, showing promising outcomes in differentiating glaucoma patients from normal controls [6,7]. Moreover, a longitudinal study found that the PERG of a glaucoma suspect (GS) displayed longitudinal loss of signal [8] and had been changed four years before VF started to show the defects [9].

OCT angiography (OCTA), which is a recently introduced device, noninvasively measures the retinal vessel density (VD) in various regions around the peripapillary and macular region [10,11]. Studies using OCTA have reported decreased peripapillary and macular VD in glaucomatous eyes compared to healthy eyes [11,12,13,14,15]. It has been shown that OCTA also has a promising possibility in monitoring glaucoma progression in some studies [16,17,18,19].

There has been a lack of studies evaluating the association between the PERG and OCTA. Therefore, the purpose of this study is to evaluate the relationship between parameters of the PERG and OCTA in open-angle glaucoma (OAG). This study also examined the association between parameters of OCT, VF, and the PERG.

## 2. Methods

### 2.1. Patients

This retrospective cross-sectional study was performed according to the tenets of the Declaration of Helsinki and approved retrospectively by the institutional review board of Yonsei University (4-2020-0780). The requirement for informed consent was waived because of the retrospective nature of the study. We reviewed the medical records of all the patients who visited the glaucoma clinic of Severance hospital from January 2017 to July 2020.

A total of 95 OAG patients and 102 normal controls were included in this study. Normal controls were people who visited our ophthalmology clinic for an ocular check-up. All the patients underwent ophthalmologic examination including slit-lamp biomicroscopy, Goldmann applanation tonometry, gonioscopy, dilated fundus examination, measurement of best-corrected visual acuity (BCVA), and measurement of axial length (AXL) (IOL Master 700; Carl Zeiss Meditec, Dublin, CA, USA). Spectral-domain OCT (Cirrus HD-OCT, software v11.0; Carl Zeiss Meditec, Dublin, CA, USA), OCTA (Cirrus-AngioPlex; Zeiss Medical Technology, Dublin, CA, USA), VF test (Humphrey Field Analyzer II; Carl Zeiss Meditec, Dublin, CA, USA), and the PERG (Neuro-ERG; Neurosoft, Ivanovo, Russia) were performed to evaluate structural and functional glaucomatous change. All the examinations were performed in one day. If retesting was needed owing to poor quality results, the tests were repeated within one month.

Inclusion criteria were as follows: (1) age ≥ 18 years old, (2) open-angle on gonioscopy, (3) BCVA ≥ 20/40, and (4) spherical equivalent (SE) within ±6.0 diopters and astigmatism within ±1.5 diopters. All OAG patients had glaucomatous optic disc (increased cup/disc ratio with localized loss or thinning of neuroretinal rim, generalized loss of disc rim, or peripapillary disc hemorrhage) and VF defects. Glaucomatous VF defects were defined as the glaucoma hemifield test was outside normal limits at either ≥3 adjacent points with *p* < 0.05 or ≥ 2 adjacent points with *p* < 0.02 on a pattern deviation probability map. We did not divide patients according to intraocular pressure (IOP), for the classification of normal-tension glaucoma (NTG) and high-tension glaucoma. Normal controls did not have glaucomatous optic discs or VF defects and showed normal retinal nerve fiber layer thickness (RNFL), which was within 95% of the internally set database in OCT results.

Patients with poor data quality or other ocular comorbidities were excluded: (1) OCTA images with artifacts or signal strength less than 7, (2) eyes with a diagnosis of pigment dispersion glaucoma, pseudoexfoliative glaucoma, or primary angle-closure glaucoma, (3) retinal disorder (diabetic retinopathy, age-related macular degeneration, retinal detachment, etc.), (4) history of ocular trauma or intraocular surgery except for uncomplicated cataract surgery at least one year before the examination, (5) history of brain disorder (brain hemorrhage, brain infarction, brain aneurysm, brain tumor, etc.), and (6) history of optic nerve disorder (optic neuritis, ischemic optic neuropathy, optic coloboma, etc.). If both eyes were eligible, one eye was randomly selected.

### 2.2. Pattern Electroretinogram

The PERG was performed by one trained examiner using a commercial ERG stimulator (Neuro-ERG; Neurosoft, Ivanovo, Russia), a device that complies with the standards set by the International Society of Clinical Electrophysiology and Vision (ISCEV) [20]. The patients without dilation of pupils were seated in a dim room with a background illumination of 50. A total of 4 electrodes were applied for stimulation. Two 35 mm Ag/AgCl skin electrodes were attached to the lower lids, and two ground electrodes were attached to both earlobes. Black and white checkerboards with a check size of 1.81° were presented on a 24 inch-monitor with a 48° × 33° visual angle, and at a distance of 60 cm. Stimuli were modulated in counterphase at 4 Hz. The checkerboards had a mean luminance of 100 cd/m^2^. Participants fixed their views at the center of the monitor, where a red-colored fixed point was placed. The PERG was measured as binocular recordings with provided appropriate refractive correction. At least 100 readings were recorded and averaged. To investigate the reproducibility of the ERG parameters, test-retest variability was measured with 30 randomly selected measurements. The latency for N35, P50, and N95 was measured from the onset of checkerboard reversal to the peak of each component. The P50 amplitude was estimated from the trough of N35 to the peak of P50. The N95 amplitude was defined as being from the peak of P50 to the trough of N95.

### 2.3. Optical Coherence Tomography Angiography

Under pupil dilation, the OCTA (Cirrus-AngioPlex; Zeiss Medical Technology, Dublin, CA, USA) images of the peripapillary and macular areas were obtained. The optical microangiography algorithm analyzes the changes in the complex signal (both intensity and phase changes contained within sequential B-scans performed at the same position) and then generates en face microvascular images in a 6 × 6 mm region. In the 6 × 6 mm scan, there were 350 A-scans in each B-scan along the horizontal dimension, and 350 B-scans were repeated twice at each location. The boundaries of the superficial and deep retinal layers were determined automatically. The inner surface of the superficial retinal layer (SRL) was defined by the internal limiting membrane. The outer surface of the SRL was denoted by the inner plexiform layer. The segmentation software automatically detected the boundaries of the retinal layers from the structural OCT cross-sectional images by measuring the gradient of OCT signals to create SRL en face images. With the aid of innate software of AngioPlex OCTA, VD was defined as the total length of perfused vasculature per unit area in a region of measurement. The VD of the selected SRL area was represented using an ETDRS grid containing concentric circles with diameters of 1, 3, and 6 mm, which were called a center (≤1 mm), an inner (1–3 mm), an outer (3–6 mm), and a full (<6 mm) area.

### 2.4. Optical Coherence Tomography

Under pupil dilation, the OCT images of the peripapillary RNFL and macular ganglion cell-inner plexiform layer (GCIPL) were obtained with optic disc cube and macular scans, respectively, using a Cirrus HD-OCT. The optic disc cube scan produced an RNFL thickness map of 6 × 6 mm (200 × 200 pixels) in the area centered on the optic nerve head. The average peripapillary RNFL thickness was measured circularly with a diameter of 3.46 mm. The macular cube scan generated a GCIPL thickness map of 6 × 6 mm (512 × 128 pixels) in the area centered on the fovea. The average and minimum macular GCIPL thickness were measured in the annulus with inner vertical and horizontal diameters of 1 and 1.2 mm, respectively, and outer vertical and horizontal diameters of 4 and 4.8 mm, respectively. All OCT scans had a signal strength of ≥6. Scans with motion artifacts, poor centration, and segmentation errors were excluded.

### 2.5. Standard Automated Perimetry

Standard automated perimetry test was performed using the Swedish Interactive Threshold Algorithm standard 24-2 program using the Humphrey Field Analyzer II. Only reliable VF test results (false-positive errors < 15%, false-negative errors < 15%, and fixation loss < 20%) were included. All examinations were individually reviewed by two glaucoma specialists (T.L. and D.R.S.), and visual fields with rim or eyelid artifacts, evidence of inattention, fatigue effects, or abnormal results caused by a disease other than glaucoma were excluded.

### 2.6. Statistical Analysis

SPSS software (ver. 23.0; SPSS Inc., Chicago, IL, USA) was used for statistical analyses. The continuous variables were summarized as the mean and standard deviation. Categorical variables were summarized as frequencies and percentages. Differences were analyzed by the Student’s *t*-test for continuous parameters and by the Chi-square test for categorical parameters. Correlations between the PERG amplitudes and average RNFL thickness, average GCIPL thickness, VF mean deviation (MD), visual field index (VFI), and OCTA parameters were evaluated based on the Pearson correlation analysis. Partial correlation analysis was done considering confounding factors including age, sex, IOP, comorbidities (hypertension (HTN) and diabetes mellitus (DM)), and AXL. In all analyses, a *p*-value less than 0.05 was taken to indicate statistical significance.

## 3. Results

A total of 102 eyes of 102 normal controls and 95 eyes of 95 OAG patients were enrolled. Detailed information of participants and differences among groups are presented in Table 1. Demographic data was comparable between two groups, except age and BCVA. The OAG group was older (47.1 ± 15.9 and 56.9 ± 14.0 years, *p* < 0.001). Average RNFL thickness (92.3 ± 8.3 and 74.5 ± 12.0 μm), average GCIPL thickness (80.2 ± 4.5 and 70.7 ± 9.1 μm), minimum GCIPL thickness (78.2 ± 5.3 and 63.3 ± 11.6 μm), VF MD (0.0 ± 8.5 and −5.4 ± 5.4 dB), and VFI (98.2 ± 3.1 and 87.9 ± 15.9%) were worse in the OAG group (all *p* < 0.001).

**Figure 1:**
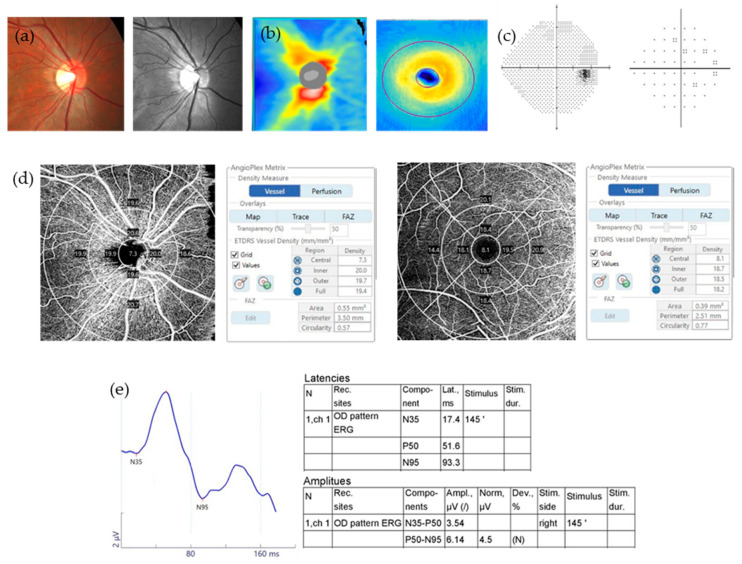
A representative case of normal controls. All examinations showed normal results. (**a**) Color and red-free fundus photography; (**b**) RNFL and GCIPL thickness of OCT; (**c**) VF; (**d**) Peripapillary VD and macular VD of OCTA; (**e**) PERG.

**Figure 2:**
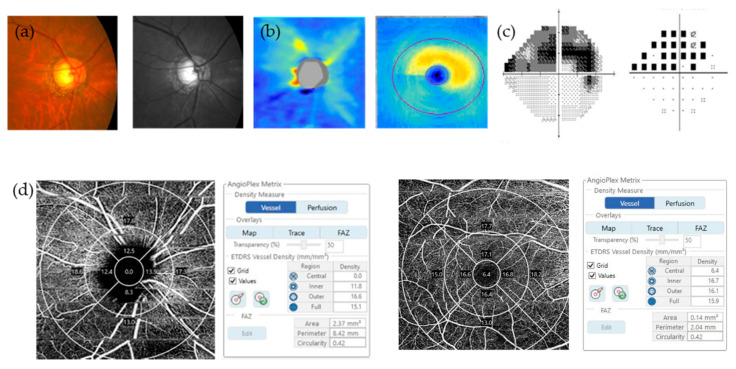

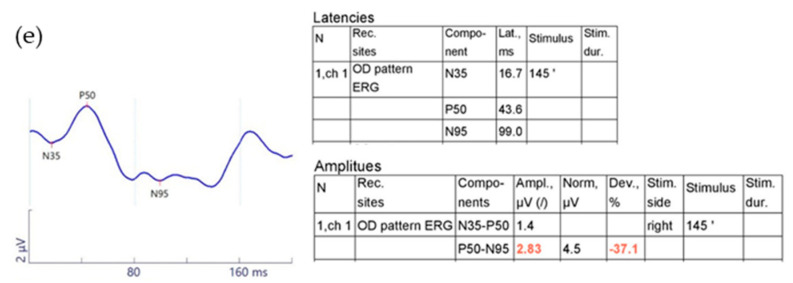
A representative case of OAG patients. (**a**) Color and red-free fundus photography; (**b**) OCT showed RNFL and GCIPL defect at inferior; (**c**) VF defect was noted at superior; (**d**) Peripapillary VD and macular VD of OCTA were reduced; (**e**) N95 amplitude of PERG was decreased to 2.83 μV. Since a reference value was set by software as 4.5 μV for N95 amplitude, results below reference value were marked red.

Table 2 shows the differences in the PERG and OCTA parameters between the two groups. In the PERG parameters, the amplitude of P50 and N95 were lower in the OAG patients (*p* = 0.011, *p* < 0.001, respectively). Regarding the OCTA parameters, all peripapillary area VDs and macular outer, full VDs were reduced in OAG patients (all *p* < 0.001). Foveal avascular zone (FAZ) area was smaller in the OAG patients (*p* = 0.015). Mean signal strength of the OCTA images of the peripapillary area (8.7 ± 1.0 and 8.6 ± 1.0) and macular area (8.7 ± 1.0 and 8.5 ± 1.0) was not significantly different between the two groups (*p* = 0.394, *p* = 0.221, respectively). Representative cases are shown in Figure 1 and Figure 2.

**Figure 3:**
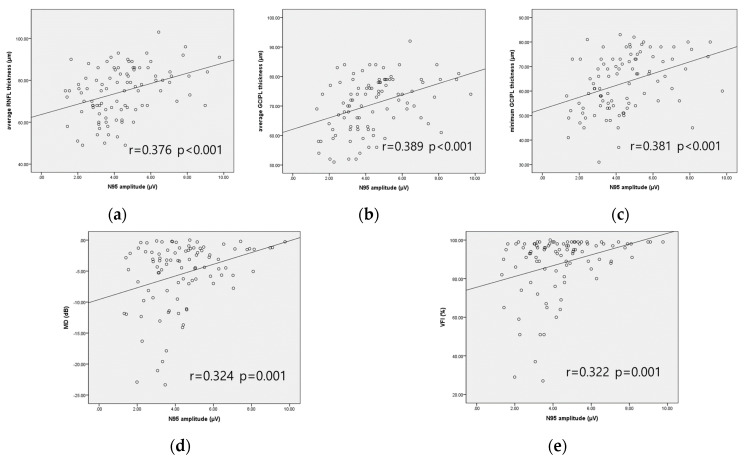
Scatterplot matrix between N95 amplitude and parameters of OCT and VF in the OAG group. N95 amplitude with (**a**) average RNFL thickness; (**b**) average GCIPL thickness; (**c**) minimum GCIPL thickness; (**d**) MD; (**e**) VFI.

**Figure 4:**
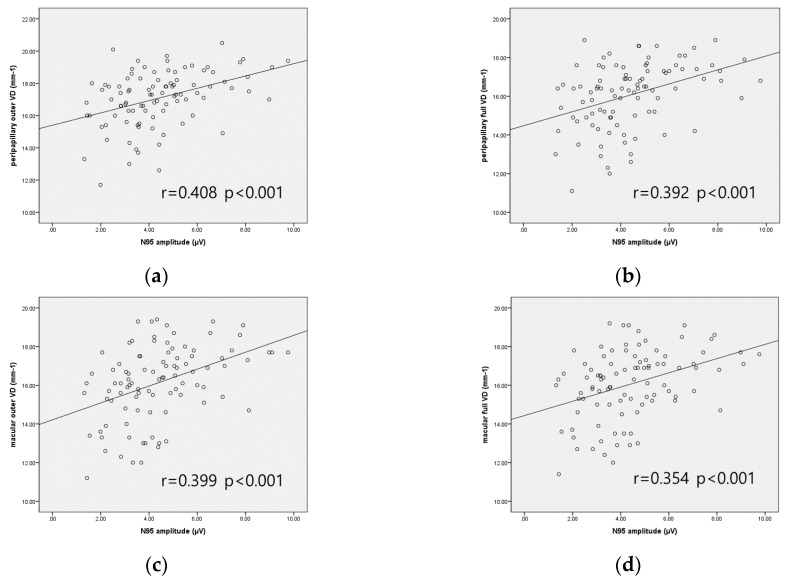
Scatterplot matrix between N95 amplitude and parameters of OCTA in OAG group. N95 amplitude with (**a**) peripapillary outer VD; (**b**) peripapillary full VD; (**c**) macular outer VD; (**d**) macular full VD.

Figure 3 and Figure 4 display the scatterplot matrix, and Table 3 shows the Pearson correlation coefficient (r) and partial correlation coefficient (r’) between N95 amplitude and parameters of OCT, VF, and OCTA in the OAG group. With Pearson correlation analysis, N95 amplitude showed significant correlation with average RNFL thickness, average and minimum GCIPL thickness, MD, VFI, peripapillary inner, outer, full VD, and macular outer, full VD. After adjusting confounding factors (age, sex, comorbidities including HTN and DM, IOP, AXL, and signal strength of OCTA), N95 amplitude was significantly positively correlated with average RNFL thickness (r’ = 0.280, *p* = 0.008), average GCIPL thickness (r’ = 0.331, *p* = 0.002), minimum GCIPL thickness (r’ = 0.338, *p* = 0.002), MD (r’ = 0.282, *p* = 0.008), VFI (r’ = 0.290, *p* = 0.006), peripapillary outer and full VD (r’ = 0.322, *p* = 0.002 and r’ = 0.279, *p* = 0.008, respectively), and macular outer and full VD (r’ = 0.339, *p* = 0.001 and r’ = 0.292, *p* = 0.006, respectively). P50 amplitude was not correlated with all parameters of VF, OCT, and OCTA except peripapillary inner VD, but correlation was not statistically significant after adjusting confounding factors (Table S1).

There were 65 eyes of 65 early-stage OAG patients whose MD was above −6 dB. Table 4 shows a subgroup analysis of early-stage OAG patients. N95 amplitude was only associated with macular outer (r’ = 0.311 *p* = 0.017) and full VD (r’ = 0.271, *p* = 0.040), not with peripapillary VD or parameters of OCT and VF.

In normal controls, neither P50 nor N95 amplitude was significantly correlated with the majority of OCTA parameters (Tables S2 and S3). Only N95 amplitude and FAZ area showed weak correlation (r’ = 0.254, *p* = 0.013).

## 4. Discussion

The result of this study revealed that N95 amplitude is positively correlated with VD measured by OCTA in OAG patients, especially of the peripapillary outer and full area and the macular outer and full area. Moreover, N95 amplitude was only associated with macular VD in early-stage OAG, not with peripapillary VD. To our knowledge, this is the first study to evaluate the relationship between parameters of the PERG and OCTA in OAG patients. Therefore, our findings provide important information regarding the relationship between decreased PERG amplitude and reduction of superficial retinal layer VD in patients with glaucoma.

The relationship of the PERG with the structural and functional change in glaucoma is controversial and various results have been published. Elgohary et al. [21] found significant differences in most PERG parameters between normal, GS, and primary open-angle glaucoma (POAG) subgroups. N95 amplitude showed a correlation with RNFL thickness, not with VF MD in POAG groups. Jeon et al. [22] showed prolonged P50 and N95 latency and decreased P50 and N95 amplitude in NTG compared to GS. P50 and N95 amplitude were significantly correlated with RNFL and GCIPL thickness in NTG. Turkey et al. [23] showed prolonged P50 and N95 latency and lower P50 and N95 amplitude in POAG and ocular hypertension (OHT). They found P50 and N95 amplitude were significantly correlated with VF parameters, but not with OCT parameters. Jung et al. [24] showed that P50 and N95 amplitude were decreased and N95 latency was prolonged in preperimetric glaucoma compared to normal controls and that N95 amplitude was significantly related with RNFL and GCIPL thickness. They even showed that N95 amplitude had superior discrimination ability than MD in the preperimetric group with RNFL defect. The diverse results are maybe due to study design, patient selection, and type of PERG examination. However, most of studies confirmed the N95 amplitude as an important parameter associated with other parameters in glaucoma. Also in our study, P50 and N95 amplitude was lower in OAG, but only N95 amplitude was significantly correlated with OCT, VF, and OCTA parameters even after adjusting confounding factors. The PERG, especially N95 amplitude, should be considered when glaucoma patients are assessed.

Although the correlation between N95 amplitude and parameters of OCT, VF, OCTA showed statistical significance, the scatterplot matrix displayed that patients with similar glaucomatous damage represented somewhat different N95 amplitudes. It is maybe due to the intrinsic variability of the PERG measurement, but since the PERG is known to represent RGC dysfunction, it is a functional measurement rather than a structural measurement. Considering a previous study which showed that the PERG predicted VF progression in GS [9], PERG parameters may differ according to disease control state or future progression possibility. We think our result is meaningful in that we showed a significant correlation between the PERG and other parameters of glaucoma including OCTA, but clinical application of the PERG should be done carefully.

Our study also showed that N95 amplitude is only associated with macular outer and full VD in early-stage OAG patients. The different result from the total OAG group is maybe mainly due to the relatively small structural damage in early-stage OAG patients. Ventura et al. [25] studied the relationship between the PERG and OCT in early glaucoma. Although they used steady-state PERG which is different from ours, they showed that the PERG reduction exceeded the proportion expected from lost RGC axons in early glaucoma. Honda et al. [26] showed that photopic negative response, which is thought to reflect inner retinal activity [27], was significantly associated with macular VD and RNFL, but not with GCIPL thickness in early NTG. Our study used N95 amplitude of transient PERG but showed relatively similar results. Considering these two previous studies, one possible explanation for our result is that microvascular alterations of the macular area affect RGC dysfunction before RGC loss causes a decrease in the GCIPL thickness or RNFL thickness in early glaucoma. Another possibility is that the blood demand of RGC decreases in cases of RGC dysfunction, but not in cases of RGC loss. Further longitudinal studies to evaluate the location of glaucomatous structural and functional progression will be needed.

Reduction of VD in the peripapillary area [10,12,28] and macular area [14,29] is well known in OAG patients, and the VD showed a more pronounced decrease as the severity of glaucoma increased [30,31]. Also, OCTA is a promising tool since it can be even useful in glaucomic eyes with high myopia [32] and in eyes with advanced glaucoma with its lower measurement floor [16]. However, it is still under debate whether the vascular changes at either optic disc or macula are a cause or a consequence of glaucomatous loss of RNFL and RGC. Our study also showed lower VD in OAG patients compared to normal controls. Superficial retinal vessels have been found to supply blood to the nerve fiber layer and ganglion cell layer, therefore reduced VD may affect RGC function, resulting in decreased PERG amplitude. To find its causal relationship, longitudinal and subgroup analysis is needed.

Usually, an OCTA scan around the optic disc is performed using a 4.5 × 4.5 mm scan, but we obtained the VD of the peripapillary area using a 6 × 6 mm scan and calculated VD according to the ETDRS circle. Since the size of the optic disc and cup is variable, subtracting the VD including and just around the optic disc may be necessary to minimize the effect of the optic disc and cup size on measurements. In our study, N95 amplitude was correlated with the peripapillary outer and full VD, not with the peripapillary center and inner VD. The macular OCTA scan is performed using a 3 × 3 mm scan or 6 × 6 mm scan. We adopted a 6 × 6 mm scan since the 6 × 6 mm scans better detect glaucomatous changes compared with the 3 × 3 mm scans [33], and N95 amplitude is again associated with the macular outer and full VD, not with the macular center and inner VD. 6 × 6 mm scan may be suitable to evaluate the VD of either peripapillary or macular area, at least considering its correlation with the PERG, but since the PERG has a minor role in glaucoma, the usefulness of 6 × 6 mm scan is needed to be analyzed in further study.

We only analyzed using the SRL of OCTA because the innate software of OCTA that we used only automatically calculated the VD of SRL, and reduction in the vasculature is reported to be more pronounced on the SRL compared with the deep retinal layer (DRL) in glaucomatous eyes [34]. The relationship of VD of DRL may further be analyzed using various methods of manually calculating VD or using other devices that automatically calculate VD of DRL.

There are several limitations to this study. First, the study is inherently limited by its retrospective design. We only included data with good image quality, therefore that selection bias might have occurred. People with old age, subsequently with cataracts and severe glaucoma were mostly excluded mainly due to poor image quality. Previous studies emphasized that OCTA data quality is influenced by signal strength, and VD differs according to signal strength [35,36,37,38]. We only included images with signal strength greater than or equal to 7 without artifacts, and two examiners (T.L and D.R.S) carefully reviewed the OCTA images and included them only when both examiners agreed on the data quality. Moreover, the mean signal strength between the two groups was not statistically different, and partial correlation analysis was done after adjusting confounding factors including signal strength of OCTA images. In addition, we performed analysis only including the images of which signal strength is 9 or 10, and the results were almost the same.

Second, the result of the study may not be applicable to the general OAG patient population. The sample in this study comprised mostly of patients with early and moderate glaucoma (87 eyes, 89.7%), whereas subjects with advanced glaucoma were fewer (10 eyes, 10.3%). However, since the PERG is a promising device for detecting early glaucomatous change in GS or OHT [3,9], the result of this study can be also meaningful for glaucoma with early to moderate stages. Further studies are needed for patients with GS or OHT.

Third, we only studied the correlation between parameters, not causal relationships or factors affecting glaucoma progression. Further longitudinal studies are needed to evaluate the PERG as a predictive factor for glaucomatous change including OCTA.

Lastly, because of its retrospective study design, the study did not take into account all confounding factors such as mean arterial pressure, heart rate, duration of DM, and glaucoma severity which had been previously identified as factors affecting the measurement of VD by OCTA. Also, IOP was measured only one time on visiting day, and we could not subdivide and analyze IOP variation such as maximum IOP, minimum IOP, IOP fluctuation, pre-treatment IOP, or post-treatment IOP. Therefore, the result of this study may be limited in a clinic situation where patients have various conditions.

## 5. Conclusions

In conclusion, N95 amplitude was correlated with structural and functional change including peripapillary and macular VD reduction in OAG patients, although the degree of correlation seemed to be weak. Microvascular alterations may be associated with dysfunctional changes of RGC recorded by the PERG in OAG.

## Figures and Tables

**Table 1:** Demographics of normal controls and OAG patients.

	Normal Group (n = 102)	OAG Group (n = 95)	*p*-Value
**Age (years)**	47.1 ± 15.9	56.9 ± 14.0	**<0.001**
Male (%)	53 (52%)	49 (51.6%)	0.957
HTN (%)	23 (22.5%)	31 (32.6%)	0.113
DM (%)	6 (5.9%)	10 (10.5%)	0.233
Pseudophakia (%)	6 (5.9%)	11 (11.6%)	0.155
**BCVA (logMAR)**	0.02 ± 0.06	0.05 ± 0.07	**0.010**
IOP (mmHg)	15.6 ± 4.6	15.5 ± 3.3	0.840
AXL (mm)	24.7 ± 1.6	24.8 ± 1.5	0.501
SE (diopter)	−1.7 ± 2.8	−1.9 ± 3.0	0.745
**Average RNFL thickness (μm)**	92.3 ± 8.3	74.5 ± 12.0	**<0.001**
**Average GCIPL thickness (μm)**	80.2 ± 4.5	70.7 ± 9.1	**<0.001**
**Minimum GCIPL thickness (μm)**	78.2 ± 5.3	63.3 ± 11.6	**<0.001**
**MD (decibel)**	0.0 ± 8.5	−5.4 ± 5.4	**<0.001**
**VFI (%)**	98.2 ± 3.1	87.9 ± 15.9	**<0.001**
Glaucoma severity (%)			
Early		65 (68.5%)	
Moderate		19 (20.0%)	
Severe		11 (10.5%)	

HTN = hypertension; DM = diabetes mellitus; BCVA = best-corrected visual acuity; IOP = intraocular pressure; AXL = axial length; SE = spherical equivalent; RNFL = retinal nerve fiber layer; GCIPL = ganglion cell-inner plexiform layer; MD = mean deviation; VFI = visual field index. All values are expressed as mean ± standard deviation. Significant differences (*p* < 0.05) are indicated as bold.

**Table 2:** Parameters of the PERG and OCTA of normal control and OAG patients.

		Normal Group (n = 102)	OAG Group (n = 95)	*p*-Value
PERG				
Latency (ms)	N35	23.6 ± 5.9	23.2 ± 7.4	0.637
	P50	48.5 ± 5.5	50.0 ± 5.3	0.051
	N95	100.6 ± 9.9	100.1 ± 12.5	0.750
**Amplitude (μV)**	**P50**	2.9 ± 1.1	2.5 ± 1.3	**0.010**
	**N95**	5.7 ± 1.7	4.4 ± 1.8	**<0.001**
OCTA				
Peripapillary				
Signal strength		8.7 ± 1.0	8.6 ± 1.0	0.394
**VD (mm^−1^)**	**Center**	2.5 ± 3.3	1.0 ± 1.6	**<0.001**
	**Inner**	16.4 ± 2.4	14.6 ± 2.7	**<0.001**
	**Outer**	18.2 ± 1.6	17.1 ± 1.7	**<0.001**
	**Full**	17.3 ± 1.4	16.1 ± 1.7	**<0.001**
Macula				
Signal strength		8.7 ± 1.0	8.5 ± 1.0	0.221
**VD (mm^−1^)**	Center	7.7 ± 2.8	8.0 ± 3.0	0.437
	Inner	17.5 ± 4.5	16.7 ± 1.8	0.144
	**Outer**	17.4 ± 1.7	16.1 ± 1.9	**<0.001**
	**Full**	17.0 ± 1.6	16.0 ± 1.8	**<0.001**
**FAZ area (mm^2^)**		0.27 ± 0.10	0.23 ± 0.11	**0.015**

VD = vessel density; FAZ = foveal avascular zone. All values are expressed as mean ± standard deviation. Significant differences (*p* < 0.05) are indicated as bold.

**Table 3:** Correlation analysis between N95 amplitude and parameters of OCT, VF, and OCTA in OAG patients.

		N95 Amplitude		After Adjusting Confounding Factors	
		r ^a^	*p*-Value	r’ ^b^	*p*-Value
**Average RNFL thickness**		0.376	<0.001	0.280	**0.008**
**Average GCIPL thickness**		0.389	<0.001	0.331	**0.002**
**Minimum GCIPL thickness**		0.381	<0.001	0.328	**0.002**
**MD**		0.324	0.001	0.282	**0.008**
**VFI**		0.322	0.001	0.290	**0.006**
**Peripapillary VD**	Center	0.130	0.209		
	Inner	0.214	0.037	0.091	0.397
	**Outer**	0.408	<0.001	0.322	**0.002**
	**Full**	0.392	<0.001	0.279	**0.008**
**Macular VD**	Center	−0.100	0.336		
	Inner	0.354	0.117		
	**Outer**	0.399	<0.001	0.339	**0.001**
	**Full**	0.354	<0.001	0.292	**0.006**
FAZ area		0.135	0.193		

Significant correlations (*p* < 0.05) are indicated as bold. ^a^ Pearson correlation coefficient. ^b^ Partial correlation coefficient (confounding factor: age, sex, HTN, DM, IOP, AXL, and signal strength of OCTA).

**Table 4:** Correlation analysis between N95 amplitude and parameters of OCT, VF, and OCTA in early-stage OAG patients.

		N95 Amplitude		After Adjusting Confounding Factors	
		r ^a^	*p*-Value	r’ ^b^	*p*-Value
Average RNFL thickness		0.295	0.017	0.126	0.344
Average GCIPL thickness		0.273	0.028	0.212	0.107
Minimum GCIPL thickness		0.237	0.057		
MD		0.065	0.605		
VFI		0.058	0.647		
Peripapillary VD	Center	0.071	0.571		
	Inner	0.079	0.533		
	Outer	0.338	0.006	0.234	0.077
	Full	0.290	0.019	0.150	0.262
**Macular VD**	Center	−0.136	0.280		
	Inner	0.161	0.201		
	**Outer**	0.376	0.002	0.311	**0.017**
	**Full**	0.345	0.005	0.271	**0.040**
FAZ area		0.108	0.391		

Significant correlations (*p* < 0.05) are indicated as bold. ^a^ Pearson correlation coefficient. ^b^ Partial correlation coefficient (confounding factor: age, sex, HTN, DM, IOP, AXL, and signal strength of OCTA).

## Supplementary Materials

The following are available online at https://www.mdpi.com/2077-0383/9/12/3854/s1, Table S1: Correlation analysis between P50 amplitude and parameters of OCT, VF, and OCTA in OAG patients, Table S2: Correlation analysis between P50 amplitude and parameters of OCT, VF, and OCTA in normal controls, and Table S3: Correlation analysis between N95 amplitude and parameters of OCT, VF, and OCTA in normal controls.
