# Supplementary material for: Relationship between N95 Amplitude of Pattern Electroretinogram and Optical Coherence Tomography Angiography in Open-Angle Glaucoma
Source: J Clin Med. 2020 Nov 27;9(12):3854. doi: 10.3390/jcm9123854 (PMC7759986; doi:10.3390/jcm9123854)
Supplement: Supplementary file 1 [file jcm-09-03854-s001.pdf]

Journal of
Clinical Medicine

MDPI

Table S1. Correlation analysis between P50 amplitude and parameters of OCT, VF, and OCTA in OAG patients.

		P50 Amplitude		After Adjusting Confounding Factors	
		r [a]	*p* Value	r′ [b]	*p* Value
Average RNFL thickness		0.099	0.341		
Average GCIPL thickness		0.178	0.085		
Minimum GCIPL thickness		0.168	0.105		
MD		0.103	0.323		
VFI		0.084	0.419		
Peripapillary VD	Center	0.114	0.269		
	Inner	0.171	0.098		
	Outer	0.220	0.033	0.020	0.854
	Full	0.233	0.023	0.046	0.671
Macular VD	Center	0.100	0.335		
	Inner	0.118	0.256		
	Outer	0.207	0.044	0.074	0.493
	Full	0.198	0.055		
FAZ area		0.025	0.809		

[a] Pearson correlation coefficient. [b] Partial correlation coefficient (confounding factor: age, sex, HTN, DM, IOP, AXL, and signal strength of OCTA).

Table S2. Correlation analysis between P50 amplitude and parameters of OCT, VF, and OCTA in normal controls.

		P50 Amplitude		After Adjusting Confounding Factors	
		r [a]	*p* Value	r′ [b]	*p* Value
Average RNFL thickness		0.099	0.323		
Average GCIPL thickness		0.192	0.053		
Minimum GCIPL thickness		0.178	0.074		
MD		0.010	0.919		
VFI		0.042	0.673		
Peripapillary VD	Center	0.121	0.225		
	Inner	−0.067	0.505		
	Outer	−0.033	0.743		
	Full	−0.045	0.654		
Macular VD	Center	−0.008	0.935		
	Inner	−0.137	0.170		
	Outer	0.085	0.396		
	Full	0.110	0.272		
FAZ area		0.157	0.115		

[a] Pearson correlation coefficient. [b] Partial correlation coefficient (confounding factor: age, sex, HTN, DM, IOP, AXL, and signal strength of OCTA).

Table S3. Correlation analysis between N95 amplitude and parameters of OCT, VF, and OCTA in normal controls.

		**N95 Amplitude**		**After Adjusting Confounding Factors**	
		**r** [a]	***p* Value**	**r′** [b]	***p* Value**
Average RNFL thickness		0.101	0.313		
Average GCIPL thickness		0.182	0.067		
Minimum GCIPL thickness		0.191	0.055		
MD		0.081	0.417		
VFI		0.023	0.820		
Peripapillary VD	Center	0.025	0.805		
	Inner	−0.048	0.631		
	Outer	0.027	0.789		
	Full	0.006	0.955		
Macular VD	Center	−0.158	0.113		
	Inner	−0.142	0.155		
	Outer	0.016	0.871		
	Full	−0.006	0.952		
**FAZ area**		0.292	0.003	0.254	**0.013**

Significant correlations ($p < 0.05$) are indicated in bold. [a] Pearson correlation coefficient. [b] Partial correlation coefficient (confounding factor: age, sex, HTN, DM, IOP, AXL, and signal strength of OCTA).
